# The Degradome database: expanding roles of mammalian proteases in life and disease

**DOI:** 10.1093/nar/gkv1201

**Published:** 2015-11-08

**Authors:** José G. Pérez-Silva, Yaiza Español, Gloria Velasco, Víctor Quesada

**Affiliations:** From the Departamento de Bioquímica y Biología Molecular, Facultad de Medicina, Instituto Universitario de Oncología, Universidad de Oviedo, 33006 Oviedo, Spain

## Abstract

Since the definition of the degradome as the complete repertoire of proteases in a given organism, the combined effort of numerous laboratories has greatly expanded our knowledge of its roles in biology and pathology. Once the genomic sequences of several important model organisms were made available, we presented the Degradome database containing the curated sets of known protease genes in human, chimpanzee, mouse and rat. Here, we describe the updated Degradome database, featuring 81 new protease genes and 7 new protease families. Notably, in this short time span, the number of known hereditary diseases caused by mutations in protease genes has increased from 77 to 119. This increase reflects the growing interest on the roles of the degradome in multiple diseases, including cancer and ageing. Finally, we have leveraged the widespread adoption of new webtools to provide interactive graphic views that show information about proteases in the global context of the degradome. The Degradome database can be accessed through its web interface at http://degradome.uniovi.es.

## INTRODUCTION

Proteases catalyze the hydrolysis of peptide bonds in a fundamentally irreversible reaction. This means that these enzymes must be tightly regulated in terms of activation and specificity to avoid massive homeostatic disorders. In turn, this need for specificity has led to the evolutionary expansion of protease genes to regulate the correct proteolysis of a large set of substrates. The parallel expansion of protease inhibitor genes added an additional level of complexity to this biochemical process. In recognition of this complex and interwoven system, the degradome of an organism was defined as the complete set of proteases in that organism (1). The degradome has been shown to affect most of the characterized biochemical pathways. Thus, different proteases are known to play key roles in such biological processes as cell cycle progression, tissue remodelling, neuronal outgrowth, haemostasis, wound healing, immunity, angiogenesis and apoptosis (2–6). Conversely, failures in the regulation of the degradome underlie diverse pathological conditions, including cancer, arthritis, progeria and neurological diseases (7–10).

Since the definition of the degradome relies on a global appraisal of the proteolytic processes, it follows that degradomics, the set of techniques specifically aimed at characterizing the degradome, must manage and integrate high-throughput data. In this regard, the completion of multiple genome projects allowed researchers to extend the degradomes of several species *in silico* from known protease sequences (11,12). Our experience in degradomics led us to tackle this problem with methods that relied heavily on manual curation after automatic predictions (13). As an additional advantage of this approach, a part of these projects consisted in the mining of the literature looking for known relationships between protease alterations and hereditary diseases, termed degradomopathies.

With this information, we described the Degradome database, containing the results of the manual annotation of every protease gene in the genomes of human, chimpanzee, mouse, and rat, along with relationships between protease alterations and hereditary diseases (14). This database complemented existing databases devoted to proteases, by providing a different focus. For instance, CutDB documents actual and predicted proteolytic events, but does not provide a global view of the proteases themselves (15). Also, MEROPS is a comprehensive and excellent database which relies on large-scale experiments and automatic predictions (16). By contrast, the Degradome database relies on manual annotation and exhaustive curation of genes, in multiple cases supported by direct cloning and sequencing experiments. For this reason, multiple instances of non-functional gene expansions, which our approach has filtered out, are annotated as putative proteases in the MEROPS database. In fact, the number of putative human proteases according to MEROPS is 990, whereas the Degradome database describes 588. In exchange, the MEROPS database features a very large number of species. Finally, our emphasis in diseases adds important information on the pathological relevance of some proteases, which is not directly available in other databases. In our view, these databases taken together provide an accurate depiction of the current knowledge about degradomics.

In these last years, the field of degradomics has experienced a remarkable expansion, not so much in the number of known proteases as in the biological and pathological roles played by the degradome. Thus, almost all of the growth in the number of known proteases has developed through the inclusion of new protein families which were not previously known to display proteolytic activity. This suggests that the initial annotation of proteases in these model genomes was highly successful. However, the number of known degradomopathies has undergone a sharp increase (>50%) in the last six years. In this manuscript, we present the updates to the Degradome database, including new interactive representations which, in our opinion, provide a useful global depiction of the degradomes.

## DATABASE ACCESS

### Annotation of individual proteases

**Figure 1.**
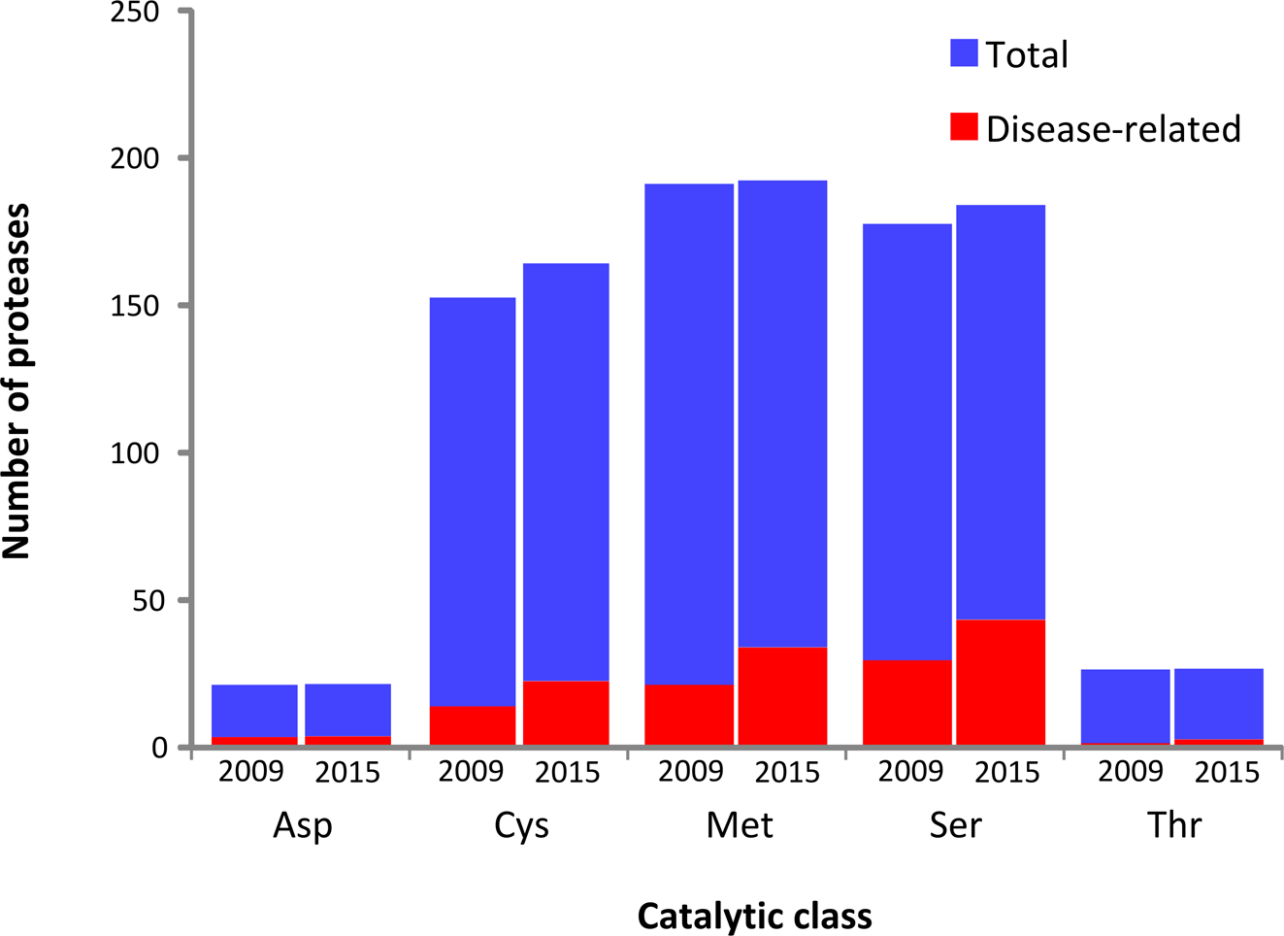
New annotations of proteases and degradomopathies. The number of proteases and degradomopathies annotated in each catalytic class is represented at the time of creation of the database (2009) and at the current version (2015).

The overall organization of the database remains unchanged, with information about five catalytic classes (aspartyl-, cysteine-, metallo-, serine- and threonine-proteases) encompassing 82 protease families in four species. Compared to the previous version of this database, we have annotated seven additional families with 18 new human and chimpanzee proteases, 21 new mouse proteases and 17 new rat proteases (Figure 1). This growth reflects a body of biochemical research by multiple laboratories which has uncovered previously unknown proteolytic activities in known proteins (17–19).

**Figure 2.**
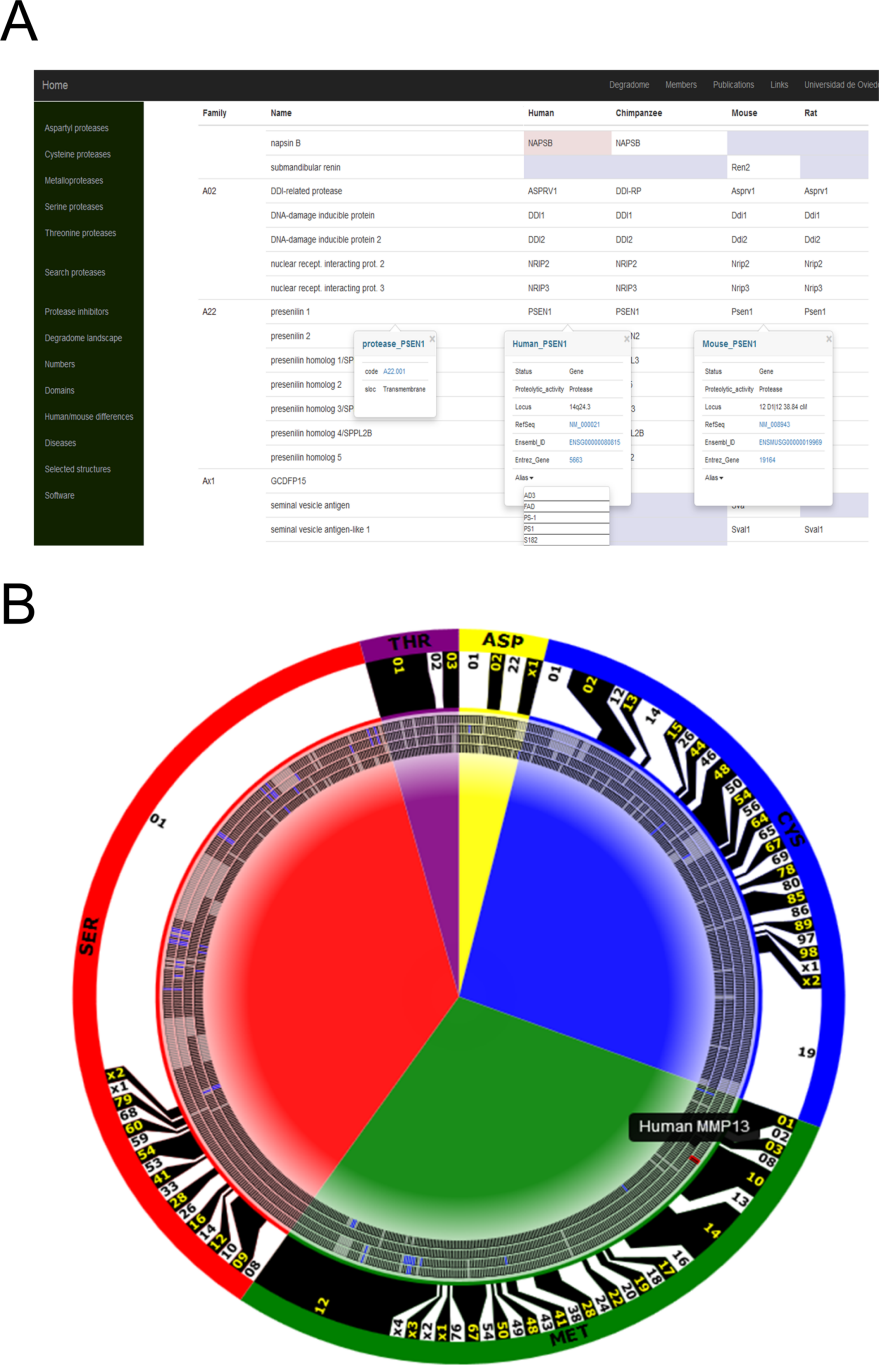
New features of the Degradome database. (**A**) Individual annotations of aspartyl proteases. The first column contains the name of the family, with a hyperlink to a web page where the user can find related selected publications and structures. The second column contains the name of each protease, with a hyperlink which opens a *popup* table with further general information—in this example, *presenilin 1*. Pseudogenes are shown over a *pink background*. Proteases absent in a species are shown as *empty grey cells*. (**B**) Interactive representation of the degradomes of (from outer to inner ticks) human, chimpanzee, mouse and rat. Protease families are limited with *black* and *white boxes*. Catalytic classes are shown as background *colored arcs*. Proteases which have been pseudogenized are depicted as *blue ticks*, and proteases absent in an organism are shown as *grey ticks*. Human collagenase-3 is highlighted to show the interactivity of the ticks.

The first mode of access is contained in five tables, one for each catalytic class. Each table displays the names of the protease families using the MEROPS classification system, the name of each protease, and the gene symbol for the protease in each species (Figure 2A). The table directly represents the orthology between proteases in different species, as well as the pseudogenes for which a functional ortholog exists in at least one of the selected mammalian species. Clicking each table cell gives the user access to additional information. Thus, the family name leads to a summary containing selected publications about that family of proteases, and, when available, a description of its structural features. In addition, protease-specific links open *popup* tables with information about the status and activity of each protease and, if available, hyperlinks to other databases, including MEROPS (16), NCBI Gene (20) and Ensembl (21). These tables also show if the protease is involved in a degradomopathy, along with a link to the OMIM database entry describing the disease.

### Hereditary diseases of proteolysis

The information about mutated proteases in hereditary diseases is kept into a separate table (http://degradome.uniovi.es/diseases.html), to bypass the need to browse or search the individual annotations. This table of degradomopathies contains information about causal gene locus, mode of inheritance, pathologic protease alteration (gain/loss of proteolytic activity), and availability of described animal models containing the same protease anomaly. A link to related OMIM entries is also provided.

To our knowledge, this remains the only summary of the relationships between degradomics and pathology. In six years, the number of entries has grown from 77 to 119, reflecting the intense research into the pathological implications of the degradome. In fact, several proteases, such as ADAM10 and AFG3L2, have been related to more than one hereditary disease through different alterations. This table does not reflect the whole contribution of the degradome to human disease, as it does not encompass the numerous examples of non-hereditary diseases in which proteases are known to play an important role through alterations in their spatio-temporal patterns of expression. In this regard, the Degradome database has also demonstrated its usefulness in the analysis of proteases associated with cancer (22,23) and ageing (4,24).

### Graphic interface

Ever since its definition, a recurrent global representation of the degradome has featured a characteristic circular drawing (Figure 2B). In this new version of the Degradome database, we have developed a tool that depicts the whole database as an svg figure. In addition to being customizable and constantly updated, this format allows a fair level of interaction with the user. Thus, each individual protease object contains a hyperlink to the corresponding annotation table. This figure can also be modified to represent the results of a search.

More importantly, this figure provides a global view of the degradomes of the included species. This not only provides a sense of the sizes of protease families, but also a fast and intuitive comparison between the degradomes of different species, including family expansions (i.e. mouse and rat expansions of the S01 family) and pseudogenization events. This interactive figure is used at this time in the home page (http://degradome.uniovi.es/dindex.html), as an alternative entry method for the database, and as a snapshot of the differences between the degradomes of human and mouse (http://degradome.uniovi.es/hmd.html).

### Additional contents

In addition to the Degradome database, the web site also keeps offering several summaries of the characteristics of mammalian degradomes. Thus, a static table listing human, mouse and rat protease inhibitors can be found at http://degradome.uniovi.es/inhibitors.html. A count of proteases in these species, itemized by catalytic class, is shown at http://degradome.uniovi.es/numbers.html. These numbers are kept updated as novel catalytic classes are discovered and added to the Degradome database. Additionally, we also keep a figure showing the different ancillary domains present in proteases (http://degradome.uniovi.es/domains.html). Interactive structures for different protease families are also kept in pdf format for teaching purposes (http://degradome.uniovi.es/structures.html).

## IMPLEMENTATION

The database with the annotations of individual proteases has been migrated to a single JSON file, which is freely available upon request. The information is queried from the web interface using the AJAX technology through JQuery. Therefore, if the browser lacks Javascript or Javascript is blocked, the user is offered a link to a static table displaying all of the information at once. The style of the web pages is implemented with the Bootstrap library to increase accessibility from multiple devices. The new graphical interface is written in svg, which is directly generated from the degradome JSON file using a custom Perl script.

Finally, *selected structures* are displayed in pdf format. Thus, the user needs Adobe Reader v7.0 or higher. Several reasons may hamper the viewing for these files from the common browsers. If this happens, the user can download the pdf file and access its contents locally.

## CONCLUSION AND FUTURE DIRECTION

The Degradome database has grown in the last years reflecting the advances in our understanding of proteases in biological and pathological processes. As a part of our involvement in degradomics, we will continue updating this database as more results become available. Based on our experience, we expect this increase to continue, driven mainly by two sources: biochemical studies that uncover novel proteolytic activities and high-throughput association studies which relate additional protease mutations with hereditary diseases.
